# Medical Colleges in Chicago

**Published:** 1871-12

**Authors:** 


### Medical Colleges in Chicago.

The Chicago Medical College, Medical Department of the Northwestern University, is located in the south part of the city, in the same block with the Mercy Hospital, and was entirely out of reach of the fire; and as the students of this College were boarding in the same section of the city, they also escaped all injury or loss. Although Professors Isham and Byford suffered severely, losing both their residences and offices, and some of the other members of the faculty suffered minor losses, yet the regular lectures in the College were suspended only one day, and even on that day while the fire was raging with all its fury, Professor E. Andrews attended his clerical hour in the hospital and performed several operations in the presence of a large part of the class. The hour for these operations had been previously fixed, and their performance, together with the resumption of the full course of lectures the following morning, was not from any want of interest in the results of the fire or in the welfare of the community, but from the conviction that one of the most efficient modes of preventing demoralization and discouragement, when some great calamity has overtaken a community, is, for every man who has anything to do and a place left to do it in, to continue diligently at his work, and employ as many others in the same way as possible. The class in attendance is fully equal to that of any previous year.

The Rush Medical College, being located in the North Division of the city was entirely consumed with all its contents. Several members of the faculty suffered very severe losses, and many of the students boarding in the vicinity also lost their books and clothing. Of course their lectures were suddenly suspended. On the next day after the fire their faculty were officially notified that all their matriculated students would be permitted to attend the lectures of the Chicago Medical College free of charge, until the faculty could procure new rooms in which to resume their lectures. And in case they did not deem it advisable to resume the present term, their students who had actually paid their lecture fees were offered the privilege of completing their course in the Chicago Medical College without further charge.

In about two weeks the Facutly effected an arrangement by which they resumed their regular course of instruction, in the amphitheatre of the County Hospital. A large part of their students returned, and so far as we know, their work is progressing satisfactorily. In the present number of the EXAMINER will be found an appeal from the Visitors of that school to its Alumni throughout the country, for aid to rebuild the College. We hope it will meet with a liberal response.

The Woman's Hospital Medical College was also burned out. Occupying temporary rooms and a recently organized institution, it had not much property to lose, and soon resumed its course of instruction in temporary quarters in the West Division of the city.

:o:

TINCTURE OF IRON IN ACUTE RHEUMATISM.—Dr. J. Russell Reynolds reports in the "British Medical Journal" eight cases of acute rheumatism successfully treated by the tinc. ferri chloridi. The pain was relieved very rapidly, and convalesence speedily established. In some of the cases the heart was implicated. The quantity given was fifty or sixty drops every six hours.—*Chicago Med. Examiner.*

